# Changes in Lifestyle and Dietary Habits during COVID-19 Lockdown in Italy: Results of an Online Survey

**DOI:** 10.3390/nu13061923

**Published:** 2021-06-03

**Authors:** Melania Prete, Anna Luzzetti, Livia S. A. Augustin, Giuseppe Porciello, Concetta Montagnese, Ilaria Calabrese, Giada Ballarin, Sergio Coluccia, Linia Patel, Sara Vitale, Elvira Palumbo, Egidio Celentano, Carlo La Vecchia, Anna Crispo

**Affiliations:** 1 Department of Clinical Science and Community Health, Università degli Studi di Milano, 20122 Milan, MI, Italy; melania.prete.1@hotmail.it (M.P.); linia.patel@unimi.it (L.P.); carlo.lavecchia@unimi.it (C.L.V.); 2 CEINGE Advanced Biotechnologies, University of Naples Federico II, Via S. Pansini 5, 80131 Naples, NA, Italy; annaluzzetti@gmail.com; 3 Epidemiology and Biostatistics Unit, Istituto Nazionale Tumori—IRCCS “Fondazione G. Pascale”, via M. Semmola 1, 80131 Naples, NA, Italy; g.porciello@istitutotumori.na.it (G.P.); sergio.coluccia@hotmail.it (S.C.); s.vitale@istitutotumori.na.it (S.V.); elvirapalumbo41@gmail.com (E.P.); e.celentano@istitutotumori.na.it (E.C.); a.crispo@istitutotumori.na.it (A.C.); 4 Department of Clinical Medicine and Surgery, Federico II University, Via S. Pansini 5, 80131 Naples, NA, Italy; ilariacalabrese@live.it; 5 Department of Movement Sciences and Wellbeing, Parthenope University, Via F. Acton 38, 80133 Naples, NA, Italy; giadaballarin@outlook.it

**Keywords:** COVID-19, lifestyle, Mediterranean diet, physical activity, sleep quality, distress

## Abstract

COVID-19 is an unprecedented global pandemic. On 12 March 2020, a lockdown order was issued in Italy in attempt to contain the health crisis. The study aimed to assess the impact of the COVID-19 lockdown on diet, physical activity, sleep quality, and distress in an Italian cohort. An online anonymous interview, which included validated questionnaires was created to compare lifestyle habits pre- and during the lockdown. Data analysis from 604 subjects with a mean age of 29.8 years was carried out using multivariate analysis. Compared to pre-COVID-19 times, 67% of people changed their eating habits and increased consumption of foods containing added sugars. Women and men with low adherence to the Mediterranean Diet (MedDiet) were more likely to be physically inactive (*p* < 0.0001 and *p* < 0.01, respectively). Results from logistic regression showed a three times higher risk of being inactive if adherence to the MedDiet was low (*p* < 0.0001), especially in men between 26 and 35 years. Lower levels of distress were reported in males who were physically active (89%) (*p* < 0.001). Our findings may help to identify effective lifestyle interventions during restrictive conditions.

## 1. Introduction

The outbreak of COVID-19 in Italy started on 21 February 2020, with 16 confirmed cases in the town of Codogno in the Lombardy region. This number increased to 60 after only 24 h and the first deaths were reported on the same day. When the epidemic spread to several Italian regions, a total lockdown of the country was declared on March the 12th. Schools and most businesses were closed and guidelines for home confinement were issued (Phase 1). These restrictions severely limited movement and interaction of people. Regulations issued within this time only permitted movement for reasons of necessity (i.e., grocery shopping) or for medical reasons. The end of Phase 1 (on May 4) marked the beginning of Phase 2, which allowed people to leave their homes and reunite with distant family members.

The consequences of such prolonged restrictions on the population were mixed [1,2]: the aim was to reduce the spread of the infection and mortality from COVID-19 disease, however they also brought about a sudden change in people’s lives with possible consequences on the health and well-being of the community. Understanding the lifestyle implications of the measures introduced to reduce COVID-19 infections may help in making well-informed decisions on the best strategies to adopt in similar future scenarios [3]. 

Several studies from different countries (Italy, Spain, and Canada) evaluated the possible lifestyle changes during COVID-19 lockdown [4,5,6,7,8,9,10,11]. Data from two Italian studies suggest that a large part of the population did not follow healthy eating habits [8] and that there was a significant reduction in habitual physical activity [9] while others found improvements in diet quality in young people [6,10,11]. Anxiety or boredom may modify nutritional choices, leading to prefer “comfort foods” rich in sugars, which cause a temporary increase in serotonin levels with a positive effect on mood and stress reduction [12,13,14,15]. In this regard, it is important to underline that domestic confinement, by influencing the stress and anxiety levels in the general population [16] may consequently influence food choices and the level of physical activity. A poor quality diet in turn may reduce body defenses against disease [17,18].

Therefore, we investigated lifestyle habits in subjects who responded to an online survey with the aim of evaluating the impact of COVID-19 lockdown on different aspects of lifestyle (diet, physical activity, sleep quality, and distress) in young Italian adults who responded to an online survey.

## 2. Materials and Methods 

An online literature search was conducted, including PubMed, Embase, and Web of Science, to search for validated questionnaires suitable for this investigation.

Four validated questionnaires were selected to evaluate different aspects of lifestyle: diet, physical activity (PA), sleep quality, and distress. The use of these questionnaires in other studies was also considered to warrant increased validity and reliability. Details of all questionnaires are reported in the Supplementary Table S1. All questionnaires were collected in a single interview, created using the Google survey tool. The interview included a total of 65 items: 5 relating to socio-demographic characteristics, 32 relating to diet and diet changes compared to the period before the lockdown, 9 relating to PA and PA changes, 11 relating to quality of sleep and its changes, 6 related to distress and its changes, and 2 questions related to the comprehension of the interview (Table S1). Participants were recruited via social media (Facebook, WhatsApp, and Instagram). Inclusion criteria included the ability to use social media, being a resident of Italy during the lockdown, and understanding more than 75% of the interview. If all these three criteria were not met, the participants were excluded. 

A total of 612 individuals participated in the survey, however two were excluded because they were not a resident in Italy, four subjects because of difficulties in understanding the questionnaire to measure the Mediterranean Diet (QueMD), and two subjects because they reported <75% understanding of the tests. Complete data from 604 participants were used for these analyses. Data was collected a month into Phase 1 lockdown (22 April to 3 May 2020). No ethic approval or informed consent was required given the nature of the study (online survey) and that data were anonymously collected on a voluntary basis.

### 2.1. Diet

The short self-managed 15-item questionnaire QueMD was used to assess eating habits [19]. QueMD allows the frequency of the consumption of nine foods usually considered key components of the Mediterranean Diet (MedDiet), namely vegetables, olive oil, dried fruits (nuts, almond, hazelnuts), whole grains, legumes, wine, fish products, and fresh fruits to be calculated. In addition to these nine items there were other items related to the consumption of dairy products (milk and yogurt), red and processed meat, sweets and fats (butter, margarine, cooking cream) and sugary drinks. For each food items, responders could choose between five consumption levels ranging from “never or rarely” to high frequency [19]. QueMD score is based on a modified version of the method proposed by Trichopoulou et al. [20] which assigns 1 point to the consumption of the following portions of: vegetables ≥ 2/day, fresh fruit ≥ 2 /day, dried fruit ≥ 2/week, whole grains ≥ 1/day, legumes ≥ 2/week), fish ≥ 2/week, and olive oil ≥ 3 tablespoons/day, red and processed meat ≤ 3/week and 1 point for drinking 1 or 2 glasses of wine per day for men or > 0 < 1/day for women. The sum of the points obtained results in the final score ranging from 0 (lowest adherence to MedDiet) to 9 (highest adherence to MedDiet).

### 2.2. Physical Activity 

To assess physical activity the International Physical Activity Questionnaire (IPAQ) a short form was used: seven items including intensity of activity (e.g., vigorous vs. moderate), the time spent walking in the last seven days, and the time spent sitting [21]. To calculate the score, the Metabolic equivalent task (METs) of the various activities were calculated as: (i) number of days in which vigorous physical activity took place x minutes spent on vigorous activity x 8; (ii) number of days during which moderate physical activity took place x minutes spent on moderate activity x 4; (iii) number of days walked x minutes spent walking x 3.3. Total METs were calculated as the sum of Walking, Moderate, and Vigorous MET scores. If total METs were ≤700, the subject was classified as inactive, for scores between 700 and 2519 the subject was classified as moderately active, and for score ≥ 2520 the subject was classified as active.

### 2.3. Distress

Distress was measured using the six-item Kessler psychological distress scale (K6) [22], which was used for an investigation on the effects of social distancing in China [3]. This questionnaire includes six questions about a person’s emotional state. Each item is scored from 0 (never) to 4 (all the time). Adding the scores of the 6 items, a minimum score of 0 and a maximum score of 24 is obtained. Scores ≤ 8 indicate no distress, scores between 9 and 16 indicate moderate distress, and scores ≥ 17 indicate high distress.

### 2.4. Sleep Quality

Sleep quality was investigated with the Pittsburgh Sleep Quality Index (PSQI) [23], which includes 19 questions grouped into 9 items. To calculate the PSQI score, the 9 items were processed to obtain 7 component scores, each of which has a value from 0 (no difficulty) to 3 (serious difficulty). The 7 scores obtained are then added together to obtain the total score that falls in a range from 0 to 21. A lower total score corresponds to better sleep quality.

### 2.5. Data Analysis

The characteristics of subjects are described as number (*n*) and percentage (%). For each questionnaire, we compared data by gender and by levels of MedDiet adherence, physical activity, sleep quality, and distress. Student t-test was used to compare continuous variable and Chi-square test was used to compared categorical variables. Multivariate logistic regression analysis was used to investigate the association between MedDiet adherence and physical activity. Results were considered statistically significant at a *p*-value < 0.05. The R software was used for all data analysis.

## 3. Results

Participants were aged between 16 and 62 years (mean age 29.8 years), 72% were women, 79% were unmarried, 43% were students and 82% were residents of southern Italy (Table 1).

Table 2 shows that during the lockdown 63% (*n* = 381) of participants showed low adherence to the MedDiet, 61% of women and 68% of men (Table 2). In addition, 67% (*n* = 406) of participants reported changes in their eating habits compared to the period before the lockdown.

A detailed analysis of the individual responses of the QueMD (data not shown) indicated that 37% reported consumption of at least 2 portions of vegetables per day, 8.1% consumed 3–4 tablespoons of olive oil per day, 24.5% consumed at least 2 portions of fresh fruit daily, 12% consumed dried fruit (nuts, almond, peanuts) more than 4 times/week, and 71% reported legume consumption at least twice a week. Dietary changes during the lockdown (data not shown) indicated the following: increased consumption of sweets, cakes and pastry products was reported by 51% of participants and reduced consumption of dried fruit by 73% of participants. The majority (>64% of participants) reported no change in the consumption of olive oil, butter, fish, red and white meat, while 55% reported no change in the consumption of bread/pasta/rice (30% indicated increases), fresh fruit (28% indicated increases), and vegetables (27% indicated increases).

Metabolic equivalents (METs) were calculated from the IPAQ-SF questionnaire. This allowed to divide subjects into three categories: 1) active (20%; *n* = 119); 2) moderately active (41%; *n* = 248); inactive (39%; *n* = 237) (Table 2). Forty-one percent of the women and 41% of the men resulted moderately active (Table 2). Five hundred and twenty participants (86%) reported that their level of PA had changed compared to the period before the lockdown, specifically, 72% (*n* = 434) claimed to be more sedentary (Table 2).

A preliminary analysis of the responses to questionnaire K6 on distress found moderate distress in 37% (*n* = 226) of participants and high distress in 27% (*n* = 162) (Table 2). Among women, 42% showed moderate distress and 29% showed high distress. The percentage of men who reported distress was lower than in women (moderate distress = 25%; high distress = 21%). Compared to pre-Covid times, among 324 subjects who reported distress, 54% also reported feeling this way a little more often than usual, while 33% reported feeling it much more often than usual (Table 2).

Regarding sleep quality, the PSQI showed that 61% (*n* = 370) of participants had low sleep quality, especially among women (64%) (Table 2). Compared to the period before lockdown, half reported less sleep or lower quality of sleep (Table 2).

Table 3 shows the distribution of responses for levels of adherence to the MedDiet, physical activity, distress, and sleep quality. Both women and men with low adherence to the MD were more likely to be physically inactive (*p* < 0.0001 and *p* < 0.01, respectively), but did not report differences in sleep quality (Table 3).

Thirty percent of participants that reported changes in their eating habits compared to the period prior to the lockdown showed also high distress (*p* < 0.01) (Table 4). This was especially evident in women, where 32% of women who claimed to have changed dietary habits presented with higher distress levels (*p* < 0.05) (Table 4).

In Table 5 we reported physical activity levels by sleep quality and distress. In the overall sample we found that those who were physically active showed lower distress (60%; *p* < 0.008). Among active men 87% reported low distress (*p* < 0.001) while this was not observed in women.

Table 6 shows the results of the logistic regression analysis of PA level for low MedDiet adherence, where it emerged that subjects with low adherence to the MedDiet had three times higher risk of being physically inactive than those with higher adherence (OR: 3.1; 95% CI: 1.9, 5.0; *p* < 0.0001) (Table 6).

## 4. Discussion

This study is the first study that simultaneously analyzes adherence to the MedDiet, PA, quality of sleep and distress levels within a young Italian cohort during the COVID-19 induced lockdown. Results indicate that dietary and lifestyle habits were negatively impacted during the lockdown. More specifically, 63% of study participants showed low adherence to the MedDiet and 67% reported changes in their eating habits compared to the period prior to the lockdown. Specifically, there was a significant increase in the intake of foods containing added sugars and saturated fats amongst 51% of subjects. In addition, subjects with low adherence to the MedDiet showed three times greater risk of being physically inactive than those with higher adherence.

It is important to consider that in recent years the populations of the Mediterranean basin (including the Italian population) have made a slow and progressive move away from the traditional Mediterranean dietary pattern to embrace an increasingly Westernized diet. Before the COVID-19 lockdown, eating habits in the Mediterranean basin were characterized by an increasing consumption of red and processed meats, sweet drinks and refined cereals, a dietary pattern associated with an increased risk of disease [24,25]. In studies carried out via online surveys in Italy, Croatia, Spain, Chile, Colombia, and Brazil in the same period [6,10,11] showed that improved adherence to the MedDiet (or its modified version) was found in young adults. This age group, unlike younger or older people, however, tends to eat outside the home frequently; hence, home confinement induced higher frequency of home cooking, which is linked to healthier dietary habits [11].

In terms of physical activity both women and men with a low adherence to the MedDiet were more likely to be physically inactive. In those doing lower levels of physical activity, a higher level of distress was reported. Moreover, most participants reported to have poorer sleep quality when compared to the pre-lockdown period. The MedDiet, characterized by a frequent intake of olive oil, fruit, vegetables, wholegrains, legumes, fish, nuts, and a low intake of processed meat and added sugars is frequently associated with reduced risk of chronic diseases such as cancer, type 2 diabetes, and cardiovascular disease [26,27,28,29]. The MedDiet score has also been shown to be inversely associated with anxiety and depression amongst adults [30]. A reduction in adherence to the MedDiet would therefore have multiple negative consequences on both physical and mental wellbeing. Firstly, on the level of immune health, it is well established that a diet rich in vitamins and minerals play an integral role in supporting the immune system [18,26,31] Vitamins A, D, E, and zinc in particular have been implied in lower susceptibility to infections and in modulating the immune response [17,26,32,33,34,35,36]. In addition, vegetables, fruit, legumes, nuts, and olive oil are rich in antioxidants, which are linked to having anti-inflammatory and anti-viral activity [34,35,37,38]. The isothiocyanates present in cruciferous vegetables (abundant in the MedDiet) are involved in the inhibition of NF-κB, an important mediator of the inflammatory process [39,40] which can be relevant in COVID-19 where high inflammatory levels have been reported [18,41].

Secondly, a lower adherence to the MedDiet as well as higher consumption of foods high in added sugar and saturated fat imply an increase in overall caloric intake. Higher energy intakes coupled with lower physical activity have been associated with increased total body fat, and particularly abdominal fat possibly driven by insulin resistance and increased circulating inflammatory cytokines [42,43]. It has been shown that metabolic status (e.g., obesity), age and sex influence the clinical severity of COVID-19 [44]. A diet high in refined carbohydrates has been shown to increase the production of pro-inflammatory cytokines [45] and high intakes of saturated fatty acids have been associated with increased corticosterone levels and increased circulating inflammatory cytokines [46]. The MedDiet however has anti-inflammatory effects [47,48,49]. Adequate control of inflammatory markers could be important to reduce the risk of severe COVID-19 [18,47,48,49]. For its anti-inflammatory and immunomodulatory effects, several studies recommend this dietary model as a potential dietary approach during COVID-19 health emergency [50,51].

Previous research indicated that an inverse association between adherence to the MedDiet and likelihood of psychological disorders including depression, anxiety, and psychological distress exists and our results support this [52]. Our findings indicate that women were more likely to report higher levels than their male counterparts. These results are in line with other studies that have found that women were more likely to report psychological distress and anxiety during lockdown [53,54,55].

Another core pillar of physical and mental wellbeing is physical activity [56]. Thirty-nine percent of the participants in our study were inactive, and as many as 76% reported changes in their PA level by becoming more sedentary, which is plausible given the mandatory home confinement. In similar studies, an increase in sitting time [57,58] and a reduction in the time spent on physical activity have also been reported [57]. A survey in Italian university students [59] and a study in the general population [60] both using IPAQ, found an overall reduction in PA (moderate and vigorous) and a significant increase in sedentary behavior during COVID-19 lockdown.

In addition, a study on global step count before and during COVID-19 supports our findings [61]. Physical Activity Guidelines for Italians recommends for adults to do at least 120 to 300 min per week of moderate PA, or 75 to 150 min per week of vigorous PA, or a combination of these. Aerobic activity should be well distributed throughout the week. PA can alleviate oxidative stress, reduce inflammation, support a healthy immune system and is known to reduce the risk of chronic diseases such as cardiovascular disease and type 2 diabetes [62,63,64,65]. In addition, moderate and vigorous PA has been linked to a reduction in psychological distress, increase feeling of well-being, improved sleep [66,67] and possibly also lead to better adherence to the MedDiet [58,68,69]. These factors may concur to help people feel better and could be important to reduce the risk of severe COVID-19 [70]. During the COVID-19 pandemic, several studies have evaluated the impact of lockdown on many aspects of lifestyle. [3,7,71,72]. In line with our studies, collective findings indicate that eating habits, sleep quality, physical activity levels, and general wellbeing were negatively impacted during the wide-scale self-isolation period [4,6]. A recent study showed a dose-dependent increase in the risk of COVID-19 in subjects who exhibited a less healthy lifestyle, compared to people who adopted a healthy lifestyle. This appears to be related to a low-grade inflammatory state as well as a higher risk of non-communicable diseases [70]. Other research has indicated that 94% of hospitalized COVID-19 patients who have died had at least one obesity-associated comorbidity such as diabetes, hypertension, cardiovascular disease, chronic lung disease, or certain cancers [73]. In the context of COVID-19 therefore, promoting a healthy lifestyle, both in terms of diet and physical exercise is pivotal. Unhealthy lifestyle is considered a risk factor for COVID-19 hospitalization [70].

However, studies conducted in different countries showed that eating habits and PA during lockdown can be influenced by age, by type of occupation, and by different family habits (e.g., eating at home versus eating out at lunch breaks), therefore comparing study results requires attention towards the comparator group and cultural differences. Other incongruencies between study results may depend on the country-specific anti-COVID-19 restrictions imposed by local governments, e.g., in Switzerland there were no restrictions for outdoors PA and sports [6,74,75]. Furthermore, adherence to the MedDiet may also change between Italian regions, as it is generally higher in the southern than in the northern regions of Italy [76].

Findings herein must be considered in the framework of the study limitations. The data is cross sectional, and hence associations between diet quality, physical activity level and levels of distress are correlational only. The recruitment method (i.e., social media platforms including Facebook, WhatsApp, and Instagram) led to a selective proportion of the Italian population being sampled. Another limitation is the recall-bias due to the conduct of the investigation during a difficult period that could have created alterations in one’s perceptions compared to the period prior to the lockdown.

## 5. Conclusions

This is the first study to evaluate the different lifestyle components together (diet, physical activity, distress, and sleep quality) in a young Italian population during COVID-19 lockdown.

Our results show that during large-scale self-isolation to control a pandemic, low adherence to the MedDiet among young Italians is associated with a lower level of physical activity and a higher level of distress, suggesting that prolonged confinement promotes unhealthy lifestyle changes which in the long term could have a negative impact on public health and complications related to COVID-19. Although causality cannot be inferred, isolation does not seem to help maintain a healthy lifestyle. We would like to highlight the importance of taking preventive measures and suggest public health programs to promote healthy diets, physical activity, and stress management during future mass lockdowns.

## Figures and Tables

**Table 1:** General characteristics of the study participants in the total subjects and by sex.

	Women *n* = 433		Men *n* = 171		Total *n* = 604		*p* *
	Mean	SD	Mean	SD	Mean	SD	
**Age**	29.7	±10.2	30.1	±11.0	29.8	±10.4	0.65 ^†^
	***n***	**(%)**	*n*	**(%)**	***N***	**(%)**	
**Age group**							
<26	200	46.2	73	42.7	273	45.2	0.51
26–35	147	33.9	57	33.3	204	33.8	
>35	86	19.9	41	23.9	127	21.0	
Total	433	100	171	100	604	100	
**Marital Status**							
Unmarried	341	78.8	134	78.4	475	78.6	0.96
Married	18	4.2	8	4.7	26	4.3	
Divorced/Widow	74	17.1	29	16.9	103	17.1	
Total	433	100	171	100	604	100	
**Occupation**							
Students	192	44.3	68	39.8	260	43.1	0.53
Workers	141	32.6	63	36.8	204	33.8	
Other	100	23.1	40	23.4	140	23.2	
Total	433	100	171	100	604	100	
**Area of residence**							
South	364	84.0	131	76.6	495	81.5	0.09
Center/North	69	15.9	40	23.4	109	18.1	
Total	433	100	171	100	604	100	

* Chi-square test for categorical variables and ^†^ Student’s t test for continuous variables for difference between men and women; SD = standard deviation.

**Table 2:** Distribution of responses for diet, physical activity, distress, and quality of sleep and perceived changes, in the total subjects and by sex.

	Women *n* = 433		Men *n* = 171		Total *n* = 604	
	*n*	(%)	*n*	(%)	*n*	(%)
**Diet adherence ^a^**					
Low	264	61.0	117	68.4	381	63.1
High	169	39.0	54	31.6	223	36.9
Total	433	100.0	171	100.0	604	100.0
**Self-reported dietary changes**				
No	134	31.0	63	36.8	197	32.7
Yes	298	69.0	108	63.2	406	67.3
**Dietary changes**						
Reduced	174	40.2	70	40.9	244	40.4
No change	118	27.3	41	24.0	159	26.3
Increased	141	32.6	60	35.1	201	33.3
**Physical activity ^b^**						
Active	81	18.7	38	22.2	119	19.7
Moderately active	178	41.1	70	40.9	248	41.1
Inactive	174	40.2	63	36.8	237	39.2
Total	433	100.0	171	100.0	604	100.0
**Physical activity changes**					
No change	65	15.0	17	9.9	82	13.6
Less sedentary	70	16.2	16	9.4	86	14.2
More sedentary	298	68.8	138	80.7	436	72.2
Distress ^c^						
Low	124	28.6	92	53.8	216	35.8
Medium	183	42.3	43	25.1	226	37.4
High	126	29.1	36	21.1	162	26.8
Total	433	100.0	171	100.0	604	100.0
**Distress changes**						
As usual	94	21.7	60	35.1	154	25.5
Much less than usual	31	7.2	24	14.0	55	9.1
Much more than usual	88	20.3	24	14.0	112	18.5
A little less than usual	15	3.5	3	1.8	18	3.0
A little more than usual	205	47.3	60	35.1	265	43.9
**Sleep quality ^d^**						
Low	278	64.2	92	53.8	370	61.3
High	155	35.8	79	46.2	234	38.7
Total	433	100.0	171	100.0	604	100.0
**Sleep quality changes**				
No changes	103	23.8	55	32.2	158	26.2
Sleep more	87	20.1	34	19.9	121	20.0
Sleep better	14	3.2	12	7.0	26	4.3
Sleep less	56	12.9	18	10.5	74	12.3
Sleep worse	174	40.0	52	30.4	225	37.3

^a^ Adherence to the Mediterranean diet: low adherence score ≤ 5; high adherence score >5. ^b^ Physical activity level: Inactive total METs ≤ 700; Moderately active total METs 701–2519; Active total METs ≥ 2520. ^c^ Distress level: no distress score ≤ 8; Moderate distress score 9–16; distress score ≥ 17. ^d^ Sleep quality level: low sleep quality score >10; high sleep quality score ≤ 10.

**Table 3:** Distribution of responses for levels of adherence to the Mediterranean diet, physical activity, distress, and sleep quality in the total subjects and by sex.

		Mediterranean Diet Adherence ^a^													
	Women *n* = 433					Men *n* = 171					Total *n* = 604				
	Low		High		*p* *	Low		High		*p* *	Low		High		*p* *
	*n*	(%)	*n*	(%)		*n*	(%)	*n*	(%)		*n*	(%)	*n*	(%)	
**Self-reported dietary changes**													
No	83	31.6	51	30.2	0.84	39	33.3	24	44.4	0.22	122	32.1	75	33.6	0.77
Yes	180	68.4	118	69.8		78	66.7	30	55.6		258	67.9	148	66.4	
Total	263	100.0	169	100.0		117	100.0	54	100.0		380	100.0	223	100.0	
**Dietary changes**															
Reduced	105	39.8	69	40.8	0.25	52	44.4	18	33.3	**<0.01**	157	41.2	87	39.0	**0.015**
No change	66	25.0	52	30.8		20	17.1	21	38.9		86	22.6	73	32.7	
Increased	93	35.2	48	28.4		45	38.5	15	27.8		138	36.2	63	28.3	
Total	264	100.0	169	100.0		117	100.0	54	100.0		381	100.0	223	100.0	
**Physical activity ^b^**														
Active	41	15.5	40	23.7	**<0.0001**	18	15.4	20	37.0	<0.01	59	15.5	60	26.9	**<0.0001**
Moderately active	94	35.6	84	49.7		49	41.9	21	38.9		143	37.5	105	47.1	
Inactive	129	48.9	45	26.6		50	42.7	13	24.1		179	47.0	58	26.0	
Total	264	100.0	169	100.0		117	100.0	54	100.0		381	100.0	223	100.0	
**Distress ^c^**															
Low	78	29.5	46	27.2	0.84	59	50.4	33	61.1	0.32	137	36.0	79	35.4	0.79
Medium	109	41.3	74	43.8		30	25.6	13	24.1		139	36.5	87	39.0	
High	77	29.2	49	29.0		28	23.9	8	14.8		105	27.6	57	25.6	
Total	264	100.0	169	100.0		117	100.0	54	100.0		381	100.0	223	100.0	
**Sleep quality ^d^**															
Low	168	63.6	110	65.1	0.84	70	59.8	22	40.7	**<0.05**	238	62.5	132	59.2	0.28
High	96	36.4	59	34.9		47	40.2	32	59.3		143	37.5	91	40.8	
Total	264	100.0	169	100.0		117	100.0	54	100.0		381	100.0	223	100.0	

^a^ Adherence to the Mediterranean diet: low adherence score ≤ 5; high adherence score >5 (up to 9). ^b^ Physical activity level: Inactive total METs ≤ 700; Moderately active total METs ≥ 701 ≤ 2519; Active total METs ≥ 2520. ^c^ Distress level: no distress score ≤ 8; Moderate distress score 9–16; distress score ≥ 17. ^d^ Sleep quality level: low sleep quality score >10; high sleep quality score ≤ 10. * Chi-square test. Bold data indicate statistically significant *p*-value (*p* < 0.05).

**Table 4:** Distribution of responses for self-reported dietary changes to physical activity, distress, and sleep quality in total subjects and by sex.

		Self-Reported Dietary Changes													
	Women *n* = 433					Men *n* = 171					Total *n* = 604				
	No		Yes		*p* *	No		Yes		*p* *	No		Yes		*p* *
	*n*	(%)	*n*	(%)		*n*	(%)	*N*	(%)		*n*	(%)	*n*	(%)	
**Physical activity ^a^**															
Active	28	20.9	53	17.8	0.69	11	17.5	27	25.0	0.22	39	19.8	80	19.7	0.92
Moderately active	52	38.8	126	42.3		31	49.2	39	36.1		83	42.1	165	40.6	
Inactive	54	40.3	119	39.9		21	33.3	42	38.9		75	38.1	161	39.7	
Total	134	100.0	298	100.0		63	100.0	108	100.0		197	100.0	406	100.0	
**Distress ^b^**															
Low	49	36.6	75	25.2	**<0.05**	38	60.3	54	50.0	0.23	87	44.2	129	31.8	**<0.01**
Medium	54	40.3	128	43.0		16	25.4	27	25.0		70	35.5	155	38.2	
High	31	23.1	95	31.9		9	14.3	27	25.0		40	20.3	122	30.0	
Total	134	100.0	298	100.0		63	100.0	108	100.0		197	100.0	406	100.0	
**Sleep quality ^c^**															
Low	80	59.7	198	66.4	0.21	32	50.8	60	55.6	0.65	112	56.9	258	63.5	0.13
High	54	40.3	100	33.6		31	49.2	48	44.4		85	43.1	148	36.5	
Total	134	100.0	298	100.0		63	100.0	108	100.0		197	100.0	406	100.0	

^a^ Physical activity level: Inactive total METs ≤ 700; Moderately active total METs 701–2519; Active total METs ≥ 2520. ^b^ Distress level: no distress score ≤ 8; Moderate distress score 9–16; distress score ≥ 17. ^c^ Sleep quality level: low sleep quality score >10; high sleep quality score ≤ 10. * Chi-square test. Bold data indicate statistically significant *p*-value (*p* < 0.05).

**Table 5:** Correlation between physical activity and sleep quality and distress in total subjects and by sex.

		Physical Activity Level ^a^																			
			Women *n* = 433							Men *n* = 171							Total *n* = 604				
	Inactive		Moderately Active		Active		*p* *	Inactive		Moderately Active		Active		*p* *	Inactive		Moderately Active		Active		*p* *
	*n*	(%)	*n*	(%)	*n*	(%)		*n*	(%)	*n*	(%)	*n*	(%)		*n*	(%)	*n*	(%)	*n*	(%)	
**Distress ^b^**																					
Low	72	14.4	62	35.2	39	46.9	0.46	29	46.0	45	64.3	33	86.8	**<0.001**	101	42.6	107	43.5	72	59.5	**<0.008**
Medium	77	14.3	88	50	34	40.9		16	25.4	18	25.7	5	13.2		93	39.2	106	43.1	39	32.2	
High	25	14.4	26	14.8	10	12.1		18	28.6	7	10	0	0		43	18.1	33	13.4	10	8.3	
Total	174	100	176	100	83	100		63	100	70	100	38	100		237	100	246	100	121	100	
**Sleep Quality ^c^**																				
Low	148	85.1	156	88.6	68	81.9	0.32	55	87.3	67	95.7	36	94.7	0.16	203	85.7	223	90.7	104	14.1	0.27
High	26	14.9	20	11.4	15	18.1		8	12.7	3	4.3	2	5.3		34	14.3	23	9.3	17	85.9	
Total	174	100	176	100	83	100		63	100	70	100	38	100		237	100	246	100	121	100	

^a^ Physical activity level: Inactive total METs ≤ 700; Moderately active total METs 701–2519; Active total METs ≥ 2520. ^b^ Distress level: no distress score ≤ 8; Moderate distress score 9–16; distress score ≥ 17. ^c^ Sleep quality level: low sleep quality score >10; high sleep quality score ≤ 10. * t-student test. Bold data indicate statistically significant *p*-value (*p* < 0.05).

**Table 6:** Multivariate logistic regression analyses of Physical activity level for low Mediterranean diet adherence.

	Low Mediterranean Diet Adherence ^a^											
	Women *n* = 433				Men *n* = 171				Total *n* = 604			
	OR ^b^	95% CI		*p* ^c^	OR ^b^	95% CI		*p* ^c^	OR ^b^	95% CI		*p* ^c^
		Lower Limit	Upper Limit			Lower Limit	Upper Limit			Lower Limit	Upper Limit	
**Physical activity ^d^**											
Active	1(Ref ^e^)				1(Ref)				1(Ref)			
Moderately active	2.50	1.59	3.92	**<0.0001**	1.75	0.75	4.13	0.195	2.12	1.422	3.16	**<0.0001**
Inactive	2.93	1.69	5.11	**<0.0001**	4.96	1.94	12.7	**0.001**	3.14	1.953	5.03	**<0.0001**

^a^ Adherence to the Mediterranean diet: low adherence score ≤ 5; high adherence score >5. **^b^** OR= Odds Ratio adjusted for terms of age (≤25; 26–35; ≥ 36); occupation (students; workers; other); region of residence (Campania; Center-North; South-Islands); gender. **^c^** Chi-square. ^d^ Physical activity level: Inactive total METs ≤ 700; Moderately active total METs 701–2519; Active total METs ≥ 2520. ^e^ Ref = Reference category. Bold data indicate statistically significant *p*-value (*p* < 0.05).

## Data Availability

The data presented in this study are available on request from the corresponding author. The data are not publicly available due to ethical restrictions.

## Supplementary Materials

The following are available on line at: https://www.mdpi.com/article/10.3390/nu13061923/s1, Table S1. Details of the survey.
