# Supplementary material for: Changes in Lifestyle and Dietary Habits during COVID-19 Lockdown in Italy: Results of an Online Survey
Source: Nutrients. 2021 Jun 3;13(6):1923. doi: 10.3390/nu13061923 (PMC8230113; doi:10.3390/nu13061923)
Supplement: Supplementary file 1 [file nutrients-13-01923-s001.zip › nutrients-1217609-supplementary.pdf]

Supplementary Table. Details of the survey.

General questions	
Sex	□ M<br>□ F
Age	Text
Civil status	□ Single<br>□ Married<br>□ Divorced<br>□ Widow or widower
Occupation	Text
City of residence	Text
**Questionnaires**	
before proceeding it is important that the compilation method is clear	□ I understood<br>□ I did not understand

QueMD

How often do you normally consume a portion of the following foods?

1.	Whole wheat pasta or rice (80gr)	□ Never or rarely;<br>□ Less than 1 time/day;<br>□ 1 times/day;<br>□ 2 times/day;<br>□ ≥ 3 times/day.
2.	Vegetables (200gr)	□ Never or rarely;<br>□ Less than 1 time/day;<br>□ 1 times/day;<br>□ 2 times/day;<br>□ ≥ 3 times/day.
3.	Fresh fruit (150gr)	□ Never or rarely;<br>□ Less than 1 time/day;<br>□ 1 times/day;<br>□ 2 times/day;<br>□ ≥ 3 times/day.
4.	Milk and yogurt (125ml)	□ Never or rarely;<br>□ Less than 1 time/day;<br>□ 1 times/day;<br>□ 2 times/day;<br>□ ≥ 3 times/day.
5.	Whole meal bread and slices (1/2 slices=50gr)	□ Never or rarely;<br>□ Less than 1 time/day;<br>□ 1-2 times/day;<br>□ 3-4 times/day;<br>□ ≥ 5 times/day.
6.	Olive oil for cooking and seasoning (1tablespoon= 10ml)	□ Never or rarely;<br>□ Less than 1 time/day;<br>□ 1-2 times/day;<br>□ 3-4 times/day;<br>□ ≥ 5 times/day.
7.	Butter. margarine or cream for cooking (1piece=10gr)	□ Never or rarely;<br>□ Less than 1 time/day;<br>□ 1-2 times/day;<br>□ 3-4 times/day;<br>□ ≥ 5 times/day.
8.	Wine (white and red) (125ml)	□ Never or rarely;<br>□ Less than 1 time/day;<br>□ 1-2 times/day;

	□ 3-4 times/day;
	□ ≥ 5 times/day.
9. Red meat (100gr). cured meat (50gr)	□ Never or rarely;
	□ Less than 1 time/week;
	□ 1-3 times/week;
	□ 4-6 times/week;
	□ ≥7 times/week
10. White meat (100gr)	□ Never or rarely;
	□ Less than 1 time/week;
	□ 1-3 times/week;
	□ 4-6 times/week;
	□ ≥7 times/week
11. Sweet or carbonated drinks (200ml)	□ Never or rarely;
	□ Less than 1 time/week;
	□ 1-3 times/week;
	□ 4-6 times/week;
	□ ≥7 times/week
12. Sweets (100gr)	□ Never or rarely;
	□ Less than 1 time/week;
	□ 1-3 times/week;
	□ 4-6 times/week;
	□ ≥7 times/week
13. Fish (150gr). seafood (50gr)	□ Never or rarely;
	□ Less than 1 time/week;
	□ 1 time/week;
	□ 2-3 times/week;
	□ ≥4 times/week
14. Dried fruit (30gr)	□ Never or rarely;
	□ Less than 1 time/week;
	□ 1 time/week;
	□ 2-3 times/week;
	□ ≥4 times/week
15. Legumes (dried 50gr. or fresh 150gr)	□ Never or rarely;
	□ Less than 1 time/week;
	□ 1 time/week;
	□ 2-3 times/week;
	□ ≥4 times/week
Has your diet changed compared to the period prior to the lockdown?	□ Yes
	□ No

Indicate if there has been a change in the consumption of one or more food categories in the last 30 days

Pasta /rice	□ Increase □ Decrease □ No change
Bread	□ Increase □ Decrease □ No change
Fresh fruits	□ Increase □ Decrease □ No change
Dried fruits	□ Increase □ Decrease □ No change
Extravirgin Olive Oil	□ Increase □ Decrease □ No change
Butter / cream / margarine	□ Increase □ Decrease □ No change
Sweets	□ Increase □ Decrease □ No change
Red and processed meat	□ Increase □ Decrease □ No change
White meat	□ Increase □ Decrease □ No change
Fish and seafood products	□ Increase □ Decrease □ No change
Wine	□ Increase □ Decrease □ No change
Vegetables	□ Increase □ Decrease □ No change
Sweet or carbonated drinks	□ Increase □ Decrease □ No change
Legumes	□ Increase □ Decrease □ No change

Milk and yogurt	□ Increase □ Decrease □ No change

IPAQ short form

1. During the last 7 days, on how many days did you do vigorous physical activities like heavy lifting, Digging, aerobics, or fast bicycling?
   _____________days [No vigorous physical activities: Skip to question 3]
2. How much time did you usually spend doing vigorous physical activities on one of those days?
   _____________minutes
3. During the last 7 days, on how many days did you do moderate physical activities like carrying light loads, bicycling at a regular pace, or doubles tennis? Do not include walking.
   _____________days [No moderate physical activities: Skip to question 5]
4. How much time did you usually spend doing moderate physical activities on one of those days?
5. _____________minutes
6. During the last 7 days, on how many days did you walk for at least 10 minutes at a time?
   _____________days [No walking: Skip to question 7]
7. How much time did you usually spend walking on one of those days?
   _____________minutes
8. During the last 7 days, how much time did you spend sitting on a weekday?
   _____________days

Has your Physical activity level changed compared to the period prior to the lockdown?	□ Yes<br>□ No
How?	□ More sedentary<br>□ Less sedentary

Distress

1. During the last 30 days, about how often did you feel...

…nervous?	□None of the time;<br>□A little of the time;<br>□Some of the time;<br>□Most of the time;<br>□All the time
…hopeless?	□None of the time;<br>□A little of the time;<br>□Some of the time;<br>□Most of the time;<br>□All the time
…restless or fidgety?	□None of the time;<br>□A little of the time;<br>□Some of the time;<br>□Most of the time;<br>□All the time
…so sad that nothing could cheer you up?	□None of the time;<br>□A little of the time;<br>□Some of the time;<br>□Most of the time;<br>□All the time
…that everything was an effort?	□None of the time;<br>□A little of the time;<br>□Some of the time;<br>□Most of the time;<br>□All the time
…worthless?	□None of the time;<br>□A little of the time;<br>□Some of the time;

□Most of the time;
□All the time

2. Did you feel this way ...
   □ Much less often than usual;
   □ less often than usual;
   □ a little less often than usual;
   □ as usual;
   □ a little thicker than usual;
   □ much thicker than usual
3. How many days, in the last 30, have you been completely unable to work or complete normal daily activities because of how you have felt? _____________days
4. Without counting those you indicated in the previous answer, how many days, in the last 30, have you been able to do only half or less than normal for how you felt? _____________days
5. In the last 30 days, how many times have you contacted a doctor or other healthcare professional about how you felt? ____________ times
6. In the last 30 days, how often have these moods been caused mainly by physical health problems? _____________times

---

Sleep quality

1. When do you usually go to bed? _____________(hh:mm)
2. How long (in minutes) does it take you to fall asleep each night? _____________minutes
3. When do you usually get up in the morning? _____________(hh:mm)
4. How many hours of actual sleep do you get at night? (This may be different than the number of hours you spend in bed) _____________(hours)
5. During the past month, how often have you had trouble sleeping because you …

a) ...cannot get to sleep within 30 minutes?
   0- Not during the past month;
   1- less than once a week;
   2- once or twice a week;
   3- three or more times week.

b) …wake up in the middle of the night or early morning?
   0- Not during the past month;
   1- less than once a week;
   2- once or twice a week;
   3- three or more times week.

c) …must get up to use the bathroom?
   0- Not during the past month;
   1- less than once a week;
   2- once or twice a week;
   3- three or more times week.

d) …cannot breathe comfortably?
   0- Not during the past month;
   1- less than once a week;
   2- once or twice a week;
   3- three or more times week.

e) …cough or snore loudly?
   0- Not during the past month;
   1- less than once a week;
   2- once or twice a week;
   3- three or more times week.

f) …feel too cold?
   0- Not during the past month;
   1- less than once a week;
   2- once or twice a week;
   3- three or more times week.

g) …feel too hot?
   0- Not during the past month;
   1- less than once a week;
   2- once or twice a week;
   3- three or more times week.

h)	…have bad dreams?	0- Not during the past month;<br>1- less than once a week;<br>2- once or twice a week;<br>3- three or more times week.
i)	…have pain?	0- Not during the past month;<br>1- less than once a week;<br>2- once or twice a week;<br>3- three or more times week.
j)	… have other reasons? (describe it)	Text

6. During the past month, how often have you taken medicine (prescribed or "over the counter") to help you sleep? _____________times
7. During the past month. how often have you had trouble staying awake while driving, eating meals, or engaging in social activity? _____________times

8. During the past month, how much of a problem has it been for you to keep up enthusiasm to get things done?	0- no problems;<br>1- slight problems;<br>2- moderate problems;<br>3-severe problems.
9. During the past month, how would you rate your sleep quality overall?	0-Very good;<br>1- fairly good;<br>2- fairly bad;<br>3-very bad.
Has your Sleep Quality changed compared to the period prior to the lockdown?<br>How?	□ Yes<br>□ No<br>□ Sleep less<br>□ Sleep worse<br>□ Sleep more<br>□ Sleep better.
About the interview, I understood:	□ I understand perfectly<br>□ I understand almost everything<br>□ I didn't understand much<br>□ I do not understand.
